# Crystal structure of 2-nitro-*N*-(5-nitro-1,3-thia­zol-2-yl)benzamide

**DOI:** 10.1107/S1600536814024374

**Published:** 2014-11-12

**Authors:** Rodolfo Moreno-Fuquen, Diego F. Sánchez, Javier Ellena

**Affiliations:** a Departamento de Química, Facultad de Ciencias Naturales y Exactas, Universidad del Valle, Apartado 25360, Santiago de Cali, Colombia; b Instituto de Física de São Carlos, IFSC, Universidade de São Paulo, USP, São Carlos, SP, Brazil

**Keywords:** crystal structure, fenilbenzamidas, 5-nitro-1,3-thia­zole derivative, hydrogen bonding

## Abstract

In the title compound, C_10_H_6_N_4_O_5_S, the mean plane of the non-H atoms of the central amide fragment C—N—C(=O)—C [r.m.s. deviation = 0.0294 Å] forms dihedral angles of 12.48 (7) and 46.66 (9)° with the planes of the thia­zole and benzene rings, respectively. In the crystal, mol­ecules are linked by N—H⋯O hydrogen bonds, forming chains along [001]. In addition, weak C—H⋯O hydrogen bonds link these chains, forming a two-dimensional network, containing *R* ^4^_4_(28) ring motifs parallel to (100).

## Related literature   

For related structures, see: Bruno *et al.* (2010 ▶, 2013 ▶); Liu *et al.* (2013 ▶). For anti­viral and anti­parasitic properties of thia­zolides, see: Korba *et al.* (2008 ▶). For hydrogen-bond details, see: Nardelli (1995 ▶).
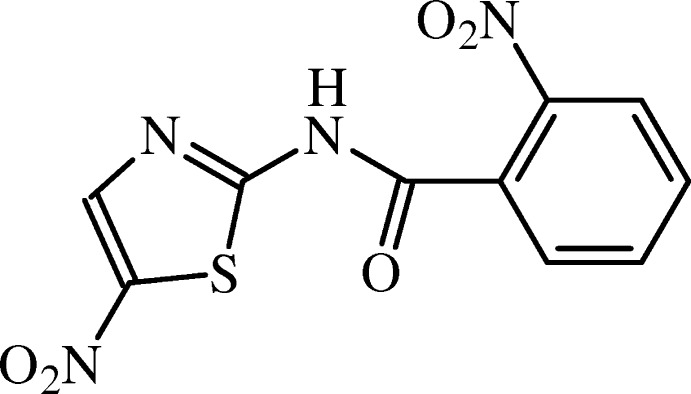


## Experimental   

### Crystal data  

C_10_H_6_N_4_O_5_S
*M* *_r_* = 294.25
Monoclinic, 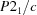

*a* = 9.6949 (2) Å
*b* = 12.4192 (2) Å
*c* = 9.8763 (2) Å
β = 94.948 (1)°
*V* = 1184.70 (4) Å^3^
*Z* = 4
Mo *K*α radiation
μ = 0.30 mm^−1^
*T* = 295 K
0.20 × 0.17 × 0.12 mm

### Data collection  

Nonius KappaCCD diffractometer
4714 measured reflections
2424 independent reflections
1867 reflections with *I* > 2σ(*I*)
*R*_int_ = 0.017

### Refinement  

*R*[*F* ^2^ > 2σ(*F* ^2^)] = 0.046
*wR*(*F* ^2^) = 0.136
*S* = 0.97
2424 reflections
181 parameters
H-atom parameters constrained
Δρ_max_ = 0.51 e Å^−3^
Δρ_min_ = −0.27 e Å^−3^

### 

Data collection: *COLLECT* (Nonius, 2000 ▶); cell refinement: *SCALEPACK* (Otwinowski & Minor, 1997 ▶); data reduction: *DENZO* (Otwinowski & Minor, 1997 ▶) and *SCALEPACK*; program(s) used to solve structure: *SHELXS97* (Sheldrick, 2008 ▶); program(s) used to refine structure: *SHELXL97* (Sheldrick, 2008 ▶); molecular graphics: *ORTEP-3 for Windows* (Farrugia, 2012 ▶) and *Mercury* (Macrae *et al.*, 2006 ▶); software used to prepare material for publication: *WinGX* (Farrugia, 2012 ▶).

## Supplementary Material

Crystal structure: contains datablock(s) I, global. DOI: 10.1107/S1600536814024374/lh5737sup1.cif

Structure factors: contains datablock(s) I. DOI: 10.1107/S1600536814024374/lh5737Isup2.hkl

Supporting information file. DOI: 10.1107/S1600536814024374/lh5737Isup3.cml

**Fig. 1.**
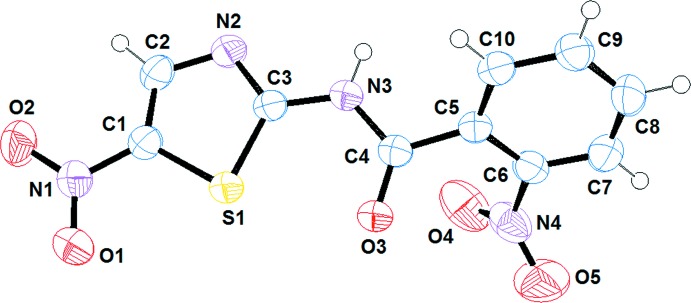
The molecular structure of (I) with displacement ellipsoids drawn at the 50% probability level. H atoms are shown as spheres of arbitrary radius.

. DOI: 10.1107/S1600536814024374/lh5737fig1.tif
The mol­ecular structure of (I) with displacement ellipsoids drawn at the 50% probability level. H atoms are shown as spheres of arbitrary radius.

**Fig. 2.**
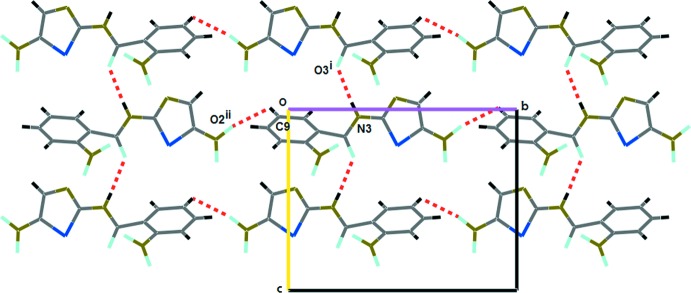
Part of the crystal structure of (I), showing the formation of R44(28) rings within a 2-D hydrogen-bonded network (dashed lines) running parallel to (100) [Symmetry codes: (i) x, -y+1/2, z-1/2; (ii) x, y-1, z].

4 4 . DOI: 10.1107/S1600536814024374/lh5737fig2.tif
Part of the crystal structure of (I), showing the formation of R^4^_4_(28) rings within a 2-D hydrogen-bonded network (dashed lines) running parallel to (100) [Symmetry codes: (i) x, −y+1/2, z-1/2; (ii) x, y-1, z].

CCDC reference: 1032923

Additional supporting information:  crystallographic information; 3D view; checkCIF report

## Figures and Tables

**Table 1:** Hydrogen-bond geometry (, )

*D*H*A*	*D*H	H*A*	*D* *A*	*D*H*A*
N3H3O3^i^	0.86	2.09	2.949(2)	175
C9H9O2^ii^	0.93	2.59	3.193(3)	123

Symmetry codes: (i) 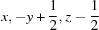; (ii) 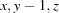.

## supplementary crystallographic information

### S1. Comment

The crystal structure determination of the title compound (I), is part of a study of a series of fenilbenzamidas derived from 5-nitro-1,3-thiazole carried out by our research group. Crystal structures of compounds similar to (I), such as 2-hydroxy-N-(5-nitro-2-thiazolyl)benzamide, (TIZ) (Bruno *et al.*, 2013), 2-acetyloxy-N-(5-nitro-2-thiazolyl)benzamide, (NTZ) (Bruno *et al.*, 2010) and N-(5-nitro-1,3-thiazol-2-yl)-4(trifluoromethyl)benzamide (NTF) (Liu *et al.*, 2013) can be compared with (I). Both the structures of (TIZ) and (NTZ) have been reported in standard antiviral essays as potent inhibitors for hepatitis B and hepatitis C replication process (Korba *et al.*, 2008). These thiazolides have also been reported as a potent antiparasitic and antiviral agents against intestinal infections caused by various parasitic protozoa (Bruno *et al.*, 2013).

The molecular structure of (I) is shown in Fig. 1. The central amide group, C3—N3—C4(═O3)—C5, is essentially planar (r.m.s. deviation for all non-H atoms = 0.0294 Å) and forms dihedral angles of 12.48 (7)° and 46.66 (9)° with the thiazole ring and benzene ring, respectively. The bond lengths and the degree of planarity in the central amide group in (I) are similar to those shown in NTZ, TIZ and NTF. The nitro O1/N1/O2 and O4/N4/O5 groups form dihedral angles of 4.07 (11)° and 47.09 (11)° with the attached thiazole and benzene rings, respectively. In the crystal (Fig. 2), molecules are connected by N—H···O hydrogen bonds and weak C—H···O hydrogen bonds (see Table 1, Nardelli, 1995). N3—H3···O3^i^ hydrogen bonds are responsible for hydrogen-bonded chains in the c-axis direction. The N3—H3 group of the amide moiety in the molecule at (x, y, z) acts as a hydrogen-bond donor to O3 atom of the carbonyl group in the molecule at (x, -y+1/2, z-1/2). In addition, weak hydrogen bonds C9—H9···O2^ii^, form chains in the b-axis direction. The C9—H9 group of the benzene ring in the molecule at (x, y, z) acts as a hydrogen bond donor to atom O2 in the molecule at (x, y-1, z). It is possible that for this weak hydrogen bond to occur, a rotation of the benzene ring with respect to plane formed by the central amide moiety is required. The combination of the above hydrogen bond interactions generate edge-fused R^4^_4_(28) rings.

### S2. Experimental

The reagents and solvents for the synthesis were obtained from the Aldrich Chemical Co., and were used without additional purification. The title molecule was synthesized using equimolar amounts of 2-nitrobenzoyl chloride (0.171g, 0.923mmol) and 5-nitro-1,3-thiazole (0.134g). The reagents were dissolved in 7 mL of acetonitrile and the solution was refluxed with constant stirring for 4 hours. A after evaporation of the solvent a brown solid was obtained. The solid was washed with distilled water to remove impurities. Pale-brown crystals of good quality [m.p. 515 (1)K] suitable for single-crystal X-ray diffraction were grown from a solution of the title compound in acetonitrile.

### S3. Refinement

All H atoms were positioned in geometrically idealized positions, with C—H = 0.93 Å and N—H = 0.86 Å, and were refined using a riding-model approximation, with *U*_iso_(H) constrained to 1.2 times *U*_eq_ of the respective parent atom.

### Crystal data

**Table:**

C_10_H_6_N_4_O_5_S	*F*(000) = 600
*M**_r_* = 294.25	*D*_x_ = 1.650 Mg m^−^^3^
Monoclinic, *P*2_1_/*c*	Melting point: 515(1) K
Hall symbol: -P 2ybc	Mo *K*α radiation, λ = 0.71073 Å
*a* = 9.6949 (2) Å	Cell parameters from 2543 reflections
*b* = 12.4192 (2) Å	θ = 2.9–26.4°
*c* = 9.8763 (2) Å	µ = 0.30 mm^−^^1^
β = 94.948 (1)°	*T* = 295 K
*V* = 1184.70 (4) Å^3^	Block, brown
*Z* = 4	0.20 × 0.17 × 0.12 mm

### Data collection

**Table:**

Nonius KappaCCD diffractometer	1867 reflections with *I* > 2σ(*I*)
Radiation source: fine-focus sealed tube	*R*_int_ = 0.017
Graphite monochromator	θ_max_ = 26.4°, θ_min_ = 3.3°
CCD rotation images, thick slices scans	*h* = −12→12
4714 measured reflections	*k* = −15→15
2424 independent reflections	*l* = −12→12

### Refinement

**Table:**

Refinement on *F*^2^	Primary atom site location: structure-invariant direct methods
Least-squares matrix: full	Secondary atom site location: difference Fourier map
*R*[*F*^2^ > 2σ(*F*^2^)] = 0.046	Hydrogen site location: inferred from neighbouring sites
*wR*(*F*^2^) = 0.136	H-atom parameters constrained
*S* = 0.97	*w* = 1/[σ^2^(*F*_o_^2^) + (0.0901*P*)^2^ + 0.3259*P*] where *P* = (*F*_o_^2^ + 2*F*_c_^2^)/3
2424 reflections	(Δ/σ)_max_ < 0.001
181 parameters	Δρ_max_ = 0.51 e Å^−^^3^
0 restraints	Δρ_min_ = −0.27 e Å^−^^3^

### Special details

**Table:**

Geometry. All esds (except the esd in the dihedral angle between two l.s. planes) are estimated using the full covariance matrix. The cell esds are taken into account individually in the estimation of esds in distances, angles and torsion angles; correlations between esds in cell parameters are only used when they are defined by crystal symmetry. An approximate (isotropic) treatment of cell esds is used for estimating esds involving l.s. planes.
Refinement. Refinement of *F*^2^ against ALL reflections. The weighted *R*-factor *wR* and goodness of fit *S* are based on *F*^2^, conventional *R*-factors *R* are based on *F*, with *F* set to zero for negative *F*^2^. The threshold expression of *F*^2^ > σ(*F*^2^) is used only for calculating *R*-factors(gt) *etc*. and is not relevant to the choice of reflections for refinement. *R*-factors based on *F*^2^ are statistically about twice as large as those based on *F*, and *R*- factors based on ALL data will be even larger.

### Fractional atomic coordinates and isotropic or equivalent isotropic displacement parameters (Å^2^)

**Table:**

	*x*	*y*	*z*	*U*_iso_*/*U*_eq_	
S1	0.33684 (6)	0.47701 (4)	0.20035 (5)	0.0441 (2)	
O3	0.22493 (17)	0.28583 (12)	0.26475 (14)	0.0502 (4)	
N1	0.4288 (2)	0.68288 (15)	0.16636 (19)	0.0473 (4)	
N2	0.3021 (2)	0.46806 (14)	−0.06343 (17)	0.0463 (5)	
N3	0.24829 (18)	0.30650 (13)	0.04092 (17)	0.0420 (4)	
H3	0.2390	0.2766	−0.0379	0.050*	
O2	0.4626 (2)	0.75643 (14)	0.09317 (19)	0.0648 (5)	
C6	0.0858 (2)	0.07681 (18)	0.1823 (2)	0.0449 (5)	
C5	0.1907 (2)	0.13054 (16)	0.12231 (19)	0.0400 (5)	
C10	0.2795 (2)	0.06972 (18)	0.0508 (2)	0.0470 (5)	
H10	0.3508	0.1032	0.0096	0.056*	
C1	0.3749 (2)	0.58660 (17)	0.1039 (2)	0.0421 (5)	
C3	0.2907 (2)	0.41287 (16)	0.04889 (19)	0.0395 (5)	
C4	0.2206 (2)	0.24675 (16)	0.1504 (2)	0.0401 (5)	
O1	0.4402 (2)	0.68685 (15)	0.29129 (17)	0.0706 (5)	
C8	0.1593 (3)	−0.09204 (19)	0.1018 (2)	0.0576 (6)	
H8	0.1498	−0.1664	0.0950	0.069*	
N4	−0.0150 (2)	0.1388 (2)	0.2531 (3)	0.0650 (6)	
C2	0.3492 (2)	0.56887 (17)	−0.0307 (2)	0.0458 (5)	
H2	0.3624	0.6212	−0.0957	0.055*	
C9	0.2627 (3)	−0.04074 (18)	0.0405 (2)	0.0536 (6)	
H9	0.3224	−0.0807	−0.0086	0.064*	
O5	−0.0429 (3)	0.1061 (2)	0.3650 (3)	0.1011 (8)	
C7	0.0691 (3)	−0.03301 (19)	0.1736 (2)	0.0545 (6)	
H7	−0.0015	−0.0670	0.2153	0.065*	
O4	−0.0647 (2)	0.2185 (2)	0.1957 (3)	0.0905 (7)	

### Atomic displacement parameters (Å^2^)

**Table:**

	*U*^11^	*U*^22^	*U*^33^	*U*^12^	*U*^13^	*U*^23^
S1	0.0603 (4)	0.0397 (3)	0.0326 (3)	−0.0048 (2)	0.0058 (2)	−0.0005 (2)
O3	0.0742 (11)	0.0441 (8)	0.0327 (8)	−0.0080 (7)	0.0074 (7)	−0.0032 (6)
N1	0.0534 (11)	0.0408 (10)	0.0477 (11)	−0.0016 (8)	0.0051 (8)	0.0001 (8)
N2	0.0615 (11)	0.0440 (10)	0.0331 (9)	−0.0017 (8)	0.0025 (8)	0.0043 (7)
N3	0.0559 (11)	0.0386 (9)	0.0318 (9)	−0.0032 (8)	0.0054 (7)	−0.0030 (7)
O2	0.0838 (13)	0.0436 (9)	0.0664 (11)	−0.0114 (8)	0.0033 (9)	0.0092 (8)
C6	0.0490 (12)	0.0471 (12)	0.0388 (11)	−0.0053 (10)	0.0044 (9)	−0.0067 (9)
C5	0.0490 (12)	0.0394 (11)	0.0313 (10)	−0.0029 (9)	0.0019 (8)	−0.0002 (8)
C10	0.0580 (13)	0.0458 (12)	0.0382 (11)	0.0012 (10)	0.0100 (10)	0.0018 (9)
C1	0.0463 (12)	0.0400 (11)	0.0403 (11)	−0.0003 (9)	0.0060 (9)	0.0015 (8)
C3	0.0453 (11)	0.0400 (11)	0.0337 (10)	0.0002 (9)	0.0061 (8)	0.0009 (8)
C4	0.0447 (11)	0.0405 (11)	0.0349 (10)	−0.0008 (9)	0.0029 (8)	−0.0020 (8)
O1	0.1054 (15)	0.0597 (11)	0.0466 (10)	−0.0183 (10)	0.0060 (9)	−0.0093 (8)
C8	0.0830 (18)	0.0375 (12)	0.0515 (14)	−0.0025 (12)	0.0019 (13)	0.0042 (10)
N4	0.0504 (12)	0.0725 (16)	0.0738 (15)	−0.0156 (11)	0.0157 (11)	−0.0284 (12)
C2	0.0539 (13)	0.0413 (12)	0.0426 (12)	−0.0009 (10)	0.0074 (10)	0.0081 (9)
C9	0.0742 (16)	0.0411 (12)	0.0464 (13)	0.0118 (11)	0.0107 (11)	0.0009 (10)
O5	0.0955 (17)	0.130 (2)	0.0857 (16)	−0.0291 (15)	0.0539 (14)	−0.0220 (14)
C7	0.0674 (15)	0.0508 (14)	0.0458 (13)	−0.0162 (11)	0.0072 (11)	0.0017 (10)
O4	0.0802 (15)	0.0786 (15)	0.1127 (18)	0.0220 (12)	0.0079 (13)	−0.0326 (14)

### Geometric parameters (Å, º)

**Table:**

S1—C3	1.720 (2)	C5—C10	1.384 (3)
S1—C1	1.720 (2)	C5—C4	1.493 (3)
O3—C4	1.227 (2)	C10—C9	1.384 (3)
N1—O2	1.227 (2)	C10—H10	0.9300
N1—O1	1.230 (2)	C1—C2	1.349 (3)
N1—C1	1.424 (3)	C8—C9	1.372 (4)
N2—C3	1.317 (2)	C8—C7	1.383 (4)
N2—C2	1.362 (3)	C8—H8	0.9300
N3—C4	1.357 (3)	N4—O4	1.218 (4)
N3—C3	1.384 (3)	N4—O5	1.230 (3)
N3—H3	0.8600	C2—H2	0.9300
C6—C7	1.375 (3)	C9—H9	0.9300
C6—C5	1.391 (3)	C7—H7	0.9300
C6—N4	1.468 (3)		
			
C3—S1—C1	86.39 (10)	N2—C3—S1	117.22 (16)
O2—N1—O1	123.74 (19)	N3—C3—S1	123.06 (15)
O2—N1—C1	118.47 (18)	O3—C4—N3	121.59 (19)
O1—N1—C1	117.78 (18)	O3—C4—C5	122.95 (18)
C3—N2—C2	109.22 (17)	N3—C4—C5	115.42 (17)
C4—N3—C3	123.71 (17)	C9—C8—C7	119.9 (2)
C4—N3—H3	118.1	C9—C8—H8	120.0
C3—N3—H3	118.1	C7—C8—H8	120.0
C7—C6—C5	122.4 (2)	O4—N4—O5	125.2 (3)
C7—C6—N4	118.1 (2)	O4—N4—C6	117.3 (2)
C5—C6—N4	119.5 (2)	O5—N4—C6	117.5 (3)
C10—C5—C6	117.7 (2)	C1—C2—N2	114.44 (18)
C10—C5—C4	120.18 (19)	C1—C2—H2	122.8
C6—C5—C4	121.46 (18)	N2—C2—H2	122.8
C5—C10—C9	120.3 (2)	C8—C9—C10	120.9 (2)
C5—C10—H10	119.9	C8—C9—H9	119.6
C9—C10—H10	119.9	C10—C9—H9	119.6
C2—C1—N1	126.41 (19)	C6—C7—C8	118.8 (2)
C2—C1—S1	112.72 (16)	C6—C7—H7	120.6
N1—C1—S1	120.87 (16)	C8—C7—H7	120.6
N2—C3—N3	119.67 (18)		
			
C7—C6—C5—C10	0.5 (3)	C3—N3—C4—O3	3.8 (3)
N4—C6—C5—C10	−176.9 (2)	C3—N3—C4—C5	−173.84 (18)
C7—C6—C5—C4	−170.5 (2)	C10—C5—C4—O3	−127.2 (2)
N4—C6—C5—C4	12.1 (3)	C6—C5—C4—O3	43.6 (3)
C6—C5—C10—C9	0.1 (3)	C10—C5—C4—N3	50.3 (3)
C4—C5—C10—C9	171.2 (2)	C6—C5—C4—N3	−138.8 (2)
O2—N1—C1—C2	−4.0 (3)	C7—C6—N4—O4	−131.6 (3)
O1—N1—C1—C2	176.9 (2)	C5—C6—N4—O4	45.9 (3)
O2—N1—C1—S1	175.42 (16)	C7—C6—N4—O5	48.1 (3)
O1—N1—C1—S1	−3.7 (3)	C5—C6—N4—O5	−134.4 (2)
C3—S1—C1—C2	1.01 (17)	N1—C1—C2—N2	177.9 (2)
C3—S1—C1—N1	−178.52 (19)	S1—C1—C2—N2	−1.6 (3)
C2—N2—C3—N3	−178.09 (18)	C3—N2—C2—C1	1.4 (3)
C2—N2—C3—S1	−0.6 (3)	C7—C8—C9—C10	0.8 (4)
C4—N3—C3—N2	−173.3 (2)	C5—C10—C9—C8	−0.7 (3)
C4—N3—C3—S1	9.4 (3)	C5—C6—C7—C8	−0.5 (3)
C1—S1—C3—N2	−0.22 (18)	N4—C6—C7—C8	177.0 (2)
C1—S1—C3—N3	177.18 (19)	C9—C8—C7—C6	−0.2 (4)

### Hydrogen-bond geometry (Å, º)

**Table:**

*D*—H···*A*	*D*—H	H···*A*	*D*···*A*	*D*—H···*A*
N3—H3···O3^i^	0.86	2.09	2.949 (2)	175
C9—H9···O2^ii^	0.93	2.59	3.193 (3)	123

Symmetry codes: (i) *x*, −*y*+1/2, *z*−1/2; (ii) *x*, *y*−1, *z*.
