# Supplementary figures and images for: Crystal structure of 2-nitro-N-(5-nitro-1,3-thia­zol-2-yl)benzamide
Source: Acta Crystallogr Sect E Struct Rep Online. 2014 Nov 12;70(Pt 12):o1252. doi: 10.1107/S1600536814024374 (PMC4257378; doi:10.1107/S1600536814024374)

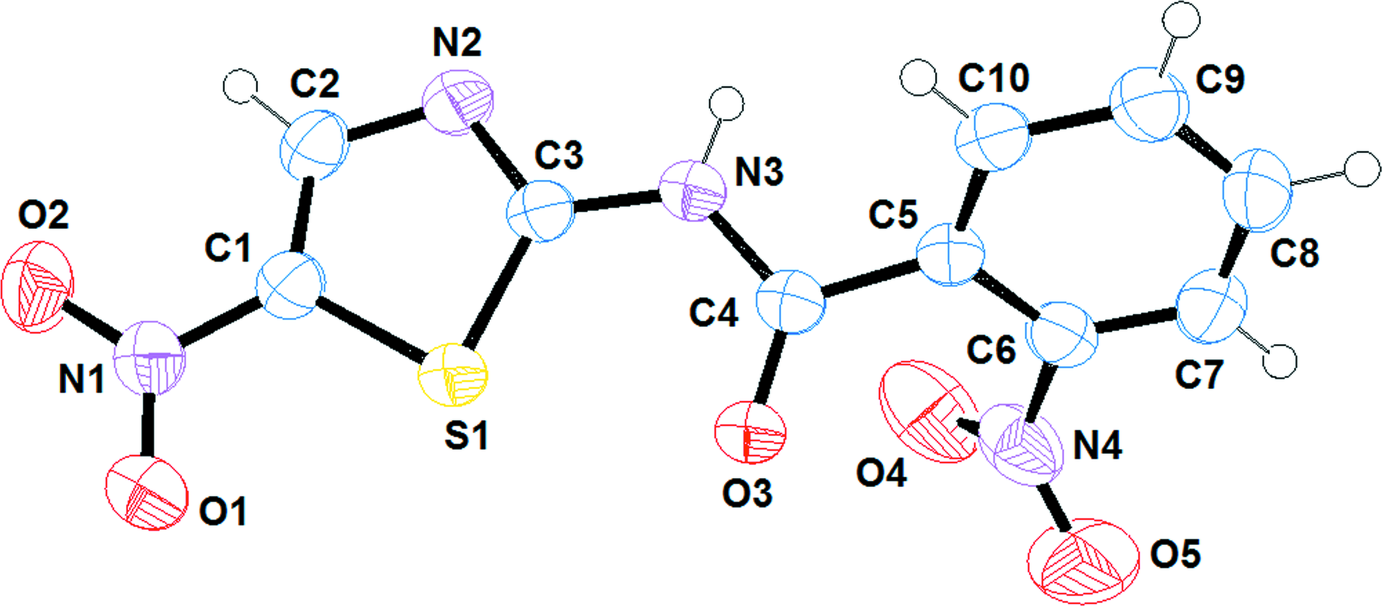

Supplement: Supplementary file 4 [file e-70-o1252-fig1.tif]

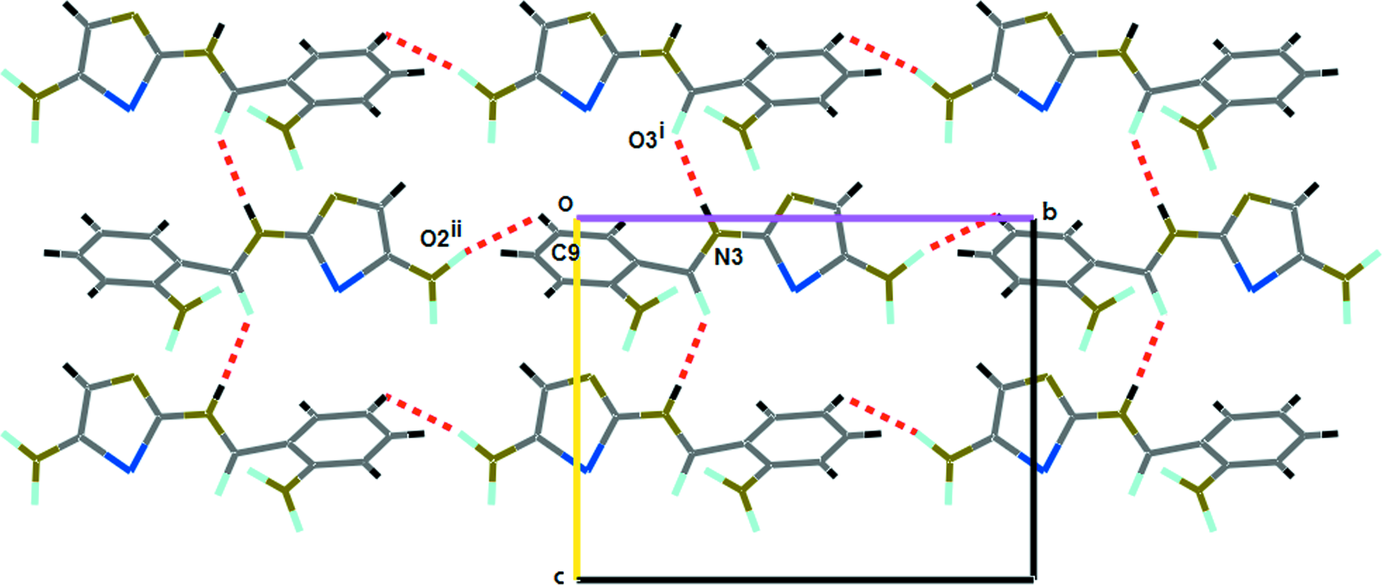

Supplement: Supplementary file 5 [file e-70-o1252-fig2.tif]
